# Supplementary material for: A set of multi-entry identification keys to African frugivorous flies (Diptera, Tephritidae)
Source: Zookeys. 2014 Jul 24;(428):97–108. doi: 10.3897/zookeys.428.7366 (PMC4143993; doi:10.3897/zookeys.428.7366)
Supplement: Supplementary material 4 — Key to Capparimyia [file zookeys-428-097-s004.zip › SF4_ZooKeys_key to Capparimyia/lucid_player/help/help/intro.htm]

Help for the Lucid3 Player


# Help for the Lucid3 Player

### Version 3.5

Welcome to the Lucid3 Player, one member of the Lucid family of products.
For more information on other Lucid and related programs, please visit
the Lucidcentral website at www.lucidcentral.org

Written in Java, Lucid3 is a cross-platform implementation, and will
run on any Java-enabled operating system (such as Windows, Macintosh,
Unix, Linux, Sun Solaris etc).

Note that the Java Virtual
Machine v1.4.2 or greater must be installed before the Lucid3 programs
will run. See the topics Java � what does
it mean? and How to check if you have
Java installed for more information.
